# Investigation of the correlation between immune thrombocytopenia and T cell activity-regulated gene polymorphism using functional study

**DOI:** 10.1038/s41598-022-10631-z

**Published:** 2022-04-22

**Authors:** Ding-Ping Chen, Wei-Tzu Lin, Ying-Hao Wen, Wei-Ting Wang

**Affiliations:** 1 Department of Laboratory Medicine, Linkou Chang Gung Memorial Hospital, Taoyuan City, 333 Taiwan, ROC; 2 Department of Medical Biotechnology and Laboratory Science, College of Medicine, Chang Gung University, Taoyuan County, Taiwan, ROC; 3 Graduate Institute of Biomedical Sciences, College of Medicine, Chang Gung University, Taoyuan County, Taiwan, ROC; 4 Graduate Institute of Clinical Medical Sciences, College of Medicine, Chang Gung University, Taoyuan, Taiwan, ROC

**Keywords:** Predictive markers, Autoimmunity

## Abstract

Thrombocytopenia is a condition where the platelet count is under 100 × 10^9^/L, which is caused by various disorders. However, the mechanism of thrombocytopenia is still unclear. Hence, we tried to investigate the correlation between immune thrombocytopenia (ITP) and single nucleotide polymorphisms (SNPs) of genes related to T cell activation. There were 32 ITP patients and 30 healthy controls enrolled in this study. PCR and sequencing were used to find out the significant SNPs, which we focused on the promoter region of CTLA4 and CD28. In this study, the ITP cases were divided into primary ITP group, secondary ITP group, and the combination of the two to the follow-up analysis. Moreover, dual-luciferase reporter assay was used to evaluate the transcription activity of the significant SNP. We found the − 1765_rs11571315 of CTLA4 gene was associated with primary ITP (p = 0.006), secondary ITP (p = 0.008), and the combination of the two (p = 0.003). Moreover, the −318_rs5742909 also had statistical significance in secondary ITP group that was only caused by autoimmune disease (p = 0.019). In functional study, the rs5742909 would decrease 19% of the transcription activity when it carried a T-allele at this position (p = 0.040). It was noted that CTLA4 gene polymorphism was related to ITP but not CD28. According to our results, we surmised that CTLA4 is involved in the pathogenesis of ITP, and the secondary ITP result from the lower CTLA4 expression that leads to T cell over-activation.

## Introduction

Platelets are derived from megakaryocytes, and their production and maturation are regulated by thrombopoietin in the bone marrow^1^. In addition to hemostasis and thrombosis, platelets are also involved in immune response and cancer biology^2^. The cut-off value of platelets in patients with thrombocytopenia is set to 100 × 10^9^/L^3^. When the counts of platelets are less than the cut-off value, it might increase the risk of bleeding, purpura and may even be life-threatening.

People with low platelet count are referred to as thrombocytopenia, which is caused by many disorders. The immune-related thrombocytopenia (ITP) may be caused by excessive destruction of platelets or inhibition of platelet production. The excessive destruction of platelets is resulted from platelet antibody (PAIgG) production^4^ or other immunological dysfunction, including type 1 helper T cells (Th1) polarization, regulatory T cells (Treg) scarcity, and autoreactive T cells activation and then leading to immune tolerance loss, megakaryocyte apoptosis, and platelet lysis^5–9^. The mechanism of over-destroyed platelets is resulted from the absence of immune-tolerance, leading to autoreactive T cells attacking their own platelets. In addition, PAIgG will adhere to the surface of platelets, which leads to platelets over-engulfed by macrophages^4,10,11^. Therefore, the differentiation and activation of immune cells play a vital role in the pathogenesis of ITP. Moreover, it is noted that B-T cell interaction is a necessary to induce ITP^12^. The costimulatory molecules, such as CTLA4 (cytotoxic T-lymphocyte-associated protein 4) and CD28, are involved in T-cell activation^13–15^, and they are the research subjects of most immune-related studies. CD28 is continually expressed on T cells^16^, and it can provide signal to promote T cell activation^17^. CTLA4 is continually expressed on Tregs or expressed on activated T cells after being induced by CD28 and T cell receptor signals^18,19^. In addition, CTLA4 would compete with CD28 for CD80/CD86 and block the stimulatory signal of T cell activation^20^. Therefore, both CTLA4 and CD28 genes were considered here.

As far as my knowledge is concerned, the mechanism of ITP is still unclear yet. In recent years, it had been shown that thrombocytopenia was associated with the transcription level of CTLA4 and regulation of T-cell activation^21,22^. Moreover, the transcription level was related to gene mutation located in the promoter region^23^. Hence, we supposed that primary ITP would be associated with abnormal immunity through T cell activity-regulated gene mutation. In this study, we investigated the correlation between single nucleotide polymorphisms (SNPs) in the promoter region of CTLA4, CD28, and ITP, and further explored the effects of the significant SNP on the gene expression. Additionally, immune-related thrombocytopenia includes primary ITP caused by unknown reason, and secondary ITP resulting from an abnormality of the bone marrow, virus infection, chemotherapy, disease, or pathological changes of liver, kidney, and spleen^24–28^, and the prevalence of thrombocytopenia caused by autoimmune disorders was less than 30%^29^. Thus, primary ITP and secondary ITP were both explored in this study.

## Results

The characteristics of patients were shown in Table 1. Five of the 32 patients with ITP were men (15.6%) and twenty-seven were women (84.4%), with a median age of 60 years old. The 11 patients were suffered from severe ITP with platelet counts below 5 × 10^9^/L. Among the ITP patients, 53.1% (17/32) cases were diagnosed as idiopathic thrombocytopenic purpura (primary ITP), and the other cases with low platelet count were caused by autoimmune disease (secondary ITP), such as systemic lupus erythematosus and rheumatoid arthritis.

**Table 1:** Characteristics of patients and controls.

	Cases, no. (%) (n = 32)	Controls, no. (%) (n = 30)
Median age of the patients	60 (56.7 ± 3.7)	22 (22.8 ± 0.3)
**Gender**		
Male	5 (15.6)	10 (33.3)
Female	27 (84.4)	20 (66.7)
**Bleeding**		
Yes	10 (31.3)	
No	22 (68.7)	
**Severity of thrombocytopenia**		
< 5 × 10^9^/L	11 (34.4)	
< 100 × 10^9^/L	21 (55.6)	
**Diagnosis**		
Primary ITP	17 (53.1)	
Secondary ITP	15 (46.9)	

Allele and genotype frequencies remain constant because of Hardy–Weinberg equilibrium (HWE) (p > 0.05) (Table 2). The allele frequency of all SNPs had no significant difference between cases and controls (Table 2). The completely statistical data of genotype frequency were shown in Table 3 and Supplementary Table S1. In association analysis of CTLA4 and CD28 gene polymorphisms with ITP (primary ITP + secondary ITP), only the genotype frequency of −1765_rs11571315 of CTLA4 had a significant difference between ITP cases and healthy controls (CC vs. CT vs. TT, p = 0.003). In addition, the control group had significantly higher heterozygous genotype frequency than ITP cases (p = 0.002).

**Table 2:** Statistical analysis of CTLA4 and CD28 allele frequency.

SNP	Allele major/minor	Minor allele frequency		HWE	Odds ratio 95% CI	*p*^a^ value
		Patient	Control	*p* value		
**CTLA4**						
−1765_rs11571315	T/C	0.129	0.183	1.000	0.660 (0.245–1.775)	0.408
−1722_rs733618	C/T	0.435	0.450	0.792	0.943 (0.461–1.927)	0.872
−1661_rs4553808	A/G	0.032	0.083	0.883	0.367 (0.068–1.968)	0.269
−1577_rs11571316	G/A	0.145	0.100	0.365	1.528 (0.509–4.593)	0.448
−1478_rs62182595	G/A	0.032	0.100	0.831	0.300 (0.058–1.550)	0.160
−1147_rs16840252	C/T	0.032	0.117	0.770	0.252 (0.050–1.268)	0.092
−318_rs5742909	C/T	0.065	0.133	0.701	0.448 (0.129–1.576)	0.202
+49_rs231775	G/A	0.333	0.283	0.449	1.204 (0.556–2.611)	0.637
CT60_rs3087643	G/A	0.222	0.150	0.897	1.619 (0.622–4.211)	0.321
**CD28**						
−1198_rs1879877	T/G	0.391	0.350	0.967	1.190 (0.573–2.471)	0.640
−1066_rs3181096	C/T	0.250	0.233	0.932	1.095 (0.481–2.495)	0.829
−1059_rs3181097	A/G	0.438	0.483	0.340	0.831 (0.410–1.687)	0.609
-1042_rs3181098	G/A	0.266	0.233	0.932	1.188 (0.526–2.687)	0.678
rs56228674	C/T	0.063	0.050	0.959	1.267 (0.271–5.910)	1.000
17+3_rs3116496	T/C	0.125	0.133	0.701	0.929 (0.325–2.654)	0.890

*HWE* Hardy–Weinberg equilibrium, *95% CI* 95% confidence interval; *p*^a^ values were counted from Chi-square test or Fisher's exact test.

**Table 3:** Statistical analysis of CTLA4 SNPs and ITP.

SNP	Genotype	Genotype frequency		Odds ratio 95% CI	*p* value
		Patient	Control		
**−1765_rs11571315**	CC vs. CT vs. TT				0.003*
T>C	TT	27	20	Ref	1.000
	CT	0	9	NA	0.002*
	CC	4	1	2.963 (0.307–28.574)	0.637
	TT vs. CT+CC			0.296 (0.081–1.083)	0.058
	TT+CT vs. CC			4.296 (0.451–40.890)	0.354
**−1722_rs733618**	CC vs. CT vs. TT				0.819
T>C	CC	12	10	Ref	1.000
	CT	11	13	0.705 (0.221–2.253)	0.555
	TT	8	7	0.952 (0.255–3.553)	0.942
	CC vs. CT+TT			0.792 (0.278–2.258)	0.662
	CC+CT vs. TT			1.143 (0.356–3.673)	0.823
**−1661_rs4553808**	AA vs. AG vs. GG				0.255
A>G/A>T	AA	29	25	Ref	1.000
	AG	2	5	345 (0.061–1.935)	0.255
	GG	0	0	NA	Na
	AA vs. AG+GG			345 (0.061–1.935)	0.255
	AA+AG vs. GG			NA	Na
**−1577_rs11571316**	AA vs. AG vs. GG				0.799
G>A/G>C	GG	24	25	Ref	1.000
	AG	5	4	1.302 (0.312–5.436)	1.000
	AA	2	1	2.083 (0.177–24.506)	1.000
	GG vs. AG+AA			1.458 (0.407–5.230)	0.561
	GG+AG vs. AA			2.000 (0.172–23.293)	1.000
**−1478_rs62182595**	AA vs. AG vs. GG				0.147
G>A	GG	29	24	Ref	1.000
	AG	2	6	0.276 (0.051–1.494)	0.147
	AA	0	0	NA	Na
	GG vs. AG+AA			0.276 (0.051–1.494)	0.147
	GG+AG vs. AA			NA	Na
**−1147_rs16840252**	CC vs. CT vs. TT				0.081
C>T	CC	29	23	Ref	1.000
	CT	2	7	0.227 (0.043–1.197)	0.081
	TT	0	0	NA	Na
	CC vs. CT+TT			0.227 (0.043–1.197)	0.081
	CC+CT vs. TT			NA	Na
**−318_rs5742909**	CC vs. CT vs. TT				0.211
C>T	CC	27	22	Ref	1.000
	CT	4	8	0.407 (0.108–1.534)	0.211
	TT	0	0	NA	Na
	CC vs. CT+TT			0.407 (0.108–1.534)	0.211
	CC+CT vs. TT			NA	Na
**+49_rs231775**	AA vs. AG vs. GG				0.157
A>G/A>T	GG	15	14	Ref	1.000
	AG	10	15	0.622 (0.211–1.836)	0.358
	AA	5	1	4.667 (0.483–45.045)	0.207
	GG vs. AG+AA			0.875 (0.318–2.410)	0.796
	GG+AG vs. AA			5.800 (0.635–53.012)	0.195
**CT60_rs3087243**	AA vs. AG vs. GG				0.516
G>A	GG	16	22	Ref	1.000
	AG	10	7	1.964 (0.615–6.271)	0.251
	AA	1	1	1.375 (0.080–23.667)	1.000
	GG vs. AG+AA			1.891 (0.620–5.768)	0.260
	GG+AG vs. AA			1.115 (0.066–18.748)	1.000

*p* values were counted from Chi-square test or Fisher's exact test, where “*” means that it had significant difference between test group and control group.

*95% CI* 95% confidence interval; *NA* not applicable.

Furthermore, the ITP cases were divided into primary ITP group without underlying disease and secondary ITP group caused by autoimmune disease to investigate their correlation with CTLA4 SNPs. We found that the rs11571315 had statistical significance in terms of primary ITP group (p = 0.006), secondary ITP group (p = 0.008), and all ITP cases (p = 0.003). Additionally, the rs11571315 also had a statistical significance when it was analyzed using the dominant model (TT vs. CT+CC), in which the p-value was 0.041 in the primary ITP group and 0.007 in the secondary ITP group. Moreover, there was only one extra significant SNP (rs5742909, p = 0.019) only in the secondary ITP caused by autoimmune disease (Table 4).

**Table 4:** The immune thrombocytopenia (ITP) cases were divided into primary ITP without underlying disease and secondary ITP caused by autoimmune disease to confirm the correlation between CTLA4 SNPs and each ITP group.

	Immune thrombocytopenia (n = 32)			Primary ITP (n = 17)			Secondary ITP (n = 15)		
	Case	Control		Case	Control		Case	Control	
**−1765_rs11571315**									
CC vs. CT vs. TT			0.003*			0.006*			0.008*
TT	27	20	1.000	13	8	1.000	14	8	1.000
CT	0	9	0.002*	0	8	0.003*	0	8	0.003*
CC	4	1	0.637	3	1	1.000	1	1	1.000
TT vs. CT+CC			0.058			0.041*			0.007*
TT+CT vs. CC			0.354			0.335			1.000
**−318_rs5742909**									
CC vs. CT vs. TT			0.211			0.708			0.019*
CC	27	22	1.000	12	11	1.000	15	11	1.000
CT	4	8	0.211	4	6	0.708	0	6	0.019*
TT	0	0	Na	0	0	Na	0	0	1.000
CC vs. CT+TT			0.211			0.708			0.019*
CC+CT vs. TT			Na			Na			1.000

“*” means that it had significant difference between test group and control group.

The same group of controls (n = 17) was used to analyze the correlation between controls and the primary ITP and secondary ITP.

We further investigated the biological effects of the significant SNPs (rs11571315 and rs5742909). In the reporter assay, C_rs11571315_ and C_rs5742909_ were considered as wild type to compare with the sequences of T_rs11571315_ and T_rs5742909_. The raw data and the bar chart were shown in supplementary raw data & Supplementary Fig. S1. The transcription activity was evaluated by calculating the luciferase ratio of NanoLuc to Firefly. Then, the luminescence value of the wild type was assumed as a reference for the relative light unit (RLU). The RLU was not different between the rs11571315 with T-allele and C-allele (p = 0.095). However, the RLU of the rs5742909 had statistical significance between the rs5742909 with T-allele and C-allele (p = 0.040). When the rs5742909 carried T-allele, it would decrease 19% of transcription activity.

## Discussion

There are various causes of thrombocytopenia, and the purpose of this study was to investigate the association between immune regulatory genes that are involved in T cell activation and ITP. The subjects with thrombocytopenia caused by organ diseases (such as lesion of liver, kidney, or spleen), drugs, chemoradiotherapy, or HIV were excluded in this research. The inclusion criteria in this study were not only limited to the low platelet counts (< 100 × 10^9^/L) without underlying disease, also known as primary ITP^30^, but also the patients suffered from thrombocytopenia triggered by autoimmune diseases were also accepted.

The promotion or inhibition of T cell activation is determined by the balance of CD28 and CTLA4 signals^31^. In addition, the SNPs located in the promoter region might affect the gene expression, and they would influence the level of protein expression and lead to the pathogenesis of disease^32^. Thus, we focused on the promoter region of CTLA4 and CD28 in this study. Based on our results, we found that the transcription activity was significantly different between rs5742909 with T-allele and C-allele in the functional assay. The previous studies also demonstrated that the gene polymorphism of rs5742909 was associated with autoimmune disease, cancer, and transfusion reaction^33–36^. Thus, patients with secondary ITP have an underlying autoimmune disease, and the polymorphisms found in the CTLA4 promoter could be related to the underlying autoimmune disease, and not necessarily to the development of ITP. Moreover, we suggested that the rs11571315 might lead to thrombocytopenia via another pathway rather than affecting CTLA4 expression.

This was the first study in the literature showed that CTLA4 gene polymorphism was related to ITP. The previous ITP-related studies only suggested that CTLA4 gene polymorphism may affect the severity of chronic thrombocytopenia^37^, or SNPs of CTLA4 were related to the mRNA transcription level. In Yao’s study, they showed that patients with primary ITP had a lower expression level of CTLA4 in Chinese Han children (5.43 ± 3.21 years old) when compared to controls, but the rs11571315 was not susceptible to primary ITP^30^. However, our data demonstrated that rs11571315 was a susceptible SNP for primary ITP in the Taiwan population. According to these two research pieces, we suggested that environmental factors may play an important role in ITP^38^, such as infection, cytokine, vaccines, and so on. These environmental factors would affect the changes of epigenetic markers that include DNA methylation, histone modification, micro-RNA regulation, etc. Zhao et al. indicated that CTLA4 plays a role in the pathogenesis of ITP as it relates to histone acetylation^39^. Additionally, it was suggested that the mechanism of ITP might be different between adults and children. This might be related to the fact that ITP in children is usually benign and self-limited, while ITP in adults is often more chronic and difficult to treat^40^. In addition, previous study showed that the rs5742909 was not associated with primary ITP in the Chinese Han population^41^, which was consistent with our results. However, we found that the gene polymorphism of rs5742909 was related to secondary ITP caused by autoimmune disease. Moreover, the rs5742909 had a significant effect on the CTLA4 expression. We could explain that it might play an important role in the development of autoimmune disease.

Our result indicated that only CTLA4 was associated with ITP but not CD28. It may be explained that CTLA4 has a higher affinity to its ligand (B7) than CD28^42^. Additionally, CTLA4 could have the ability to inhibit T-cell activation without binding B7^43^. Thus, CTLA4 had a greater impact on the pathway of T-cell activation compared to CD28. In addition, B-T (follicular helper T) cell interaction is necessary for ITP induction^12^. The main biological function of CTLA4 is to down-regulate the activity of helper T cells, and the differentiation of follicular helper T cells is controlled by CTLA4 through the participation of CD28, while the main biological function of CD28 is to modulate the activation of cytotoxic T cell^44–46^. Therefore, the effect of CTLA4 on ITP was more significant than CD28. When the amount of CTLA4 is too few to compete with CD28 for B7, the helper T cells would not be suppressed, which deeply affects the interaction between follicular helper T and B cell^20^ and then results in an autoimmune response. However, a recent study showed that the rs1980422 located downstream of CD28 was related to ITP^47^. Therefore, in the future, we should expand the region of gene to explore the SNP associated with ITP.

In conclusion, the present study indicated that CTLA4 gene polymorphism was related to the susceptibility of ITP but not CD28. Helper T cells are majorly regulated by CTLA4, which is involved in the mechanism of ITP, while CD28 is involved in cytotoxic T cell regulation. In addition, based on the results of our study and the literature^30^, we suggested that the environmental factors would also plays an important role in ITP, and the mechanism of ITP might be different between adults and children. However, the relatively small sample size was a limitation in this study, it should be verified by increasing the number of including subjects.

## Materials and methods

### Study subjects

The Institutional Review Board of Chang Gung Memorial Hospital has reviewed and approved the study. The approval ID was 201901246B0. All study subjects signed informed consent and performed in accordance with relevant guidelines and regulations. In the present study, we focused on immune thrombocytopenia, and subjects with thrombocytopenia (platelet counts less than 100 × 109/L) without receiving chemotherapy, radio therapy, bone marrow related disorder (leukemia, myelodysplastic syndromes, and aplastic anemia), liver, kidney, and spleen disorders. Seventeen of thirty-two patients were diagnosed as idiopathic thrombocytopenic purpura (ITP) in Chang Gang Memorial Hospital, while others were caused by autoimmune disorders. These inclusion criteria were based on the definition proposed by the International Working Group (IWG). Thirty healthy volunteers who had no bleeding disorders and with a normal platelet count (150 × 10^9^/L–400 × 10^9^/L) participated in this study.

### DNA extraction

The blood samples were collected in EDTA-coated vacuum tubes and genomic DNA was extracted via using QIAamp® DNA Mini kit (Qiagen GmbH, Hilden, Germany) in the light of the manufacturer’s instructions. Then, DNA concentration was assessed by measuring the optical density at 260 and 280 nm through a UV spectrometer.

### PCR amplification

The PCR mixtures were 25 µL in total, consisting of 1 µg DNA, 10 µL HotStar Taq DNA Polymerase (Qiagen GmbH, Hilden, Germany), 1 µL of forward primer (10 Mµ), 1 µL of reverse primer (10 Mµ), and 12 µL of ddH2O. The primers used in here were shown in Table 5. The PCR program for CTLA-4 primers was 1 cycle of 95 °C for 10 min, 35 cycles of 94 °C for 30 s, 65.5 °C for 30 s, and 72 °C for 1 min. The final elongation step was 3 min at 72 °C and then soaking at 10 °C forever. The PCR program for CD28 primers was 95 °C for 3 min at initial denaturation step and 30 cycles of 95 °C for 30 s, 58 °C for 30 s, and 72 °C for 2 min. The final extension step was 10 min at 72 °C and finally soaking at 10 °C indefinitely. For gel electrophoresis visualization, 5 μl of the PCR products was pipetted onto a 1.5% agarose gel and run at 100 V for 30 min. After that, the gel was visualized un-der UV illumination and ensured the correctness.

**Table 5:** Primers used for amplifying the target region of CTLA4 and CD28.

	GC content	Tm (°C)	Base pair
**primer for CTLA-4 promotor and rs231775**			
F: 5’ GGC AAC AGA GAC CCC ACC GTT 3’	21/13(62%)	65.3	1234
R: 5’ GAG GAC CTT CCT TAA ATC TGG AGA G 3’	25/12(48%)	65.8	
F: 5’ CTC TCC AGA TTT AAG GAA GGT CCT C 3’	25/12(48%)	65.8	1170
R: 5’ GGA ATA CAG AGC CAG CCA AGC C 3’	22/13(59%)	65.8	
**primer for CD28 promotor and rs3116496**			
F: 5’- GGG TGG TAA GAA TGT GGA TGA ATC-3’	24/11 (46%)	63.6	1542
R: 5’-CAA GGC ATC CTG ACT GCA GCA-3’	21/12 (57%)	63.2	
F: 5’- AAG GAT GCA GTT TAG GGT CTA GAT T -3’	25/10 (40%)	62.5	886
R: 5’-GAT CAA GCC AAC ATT GTC CAT TGG-3’	24/11 (46%)	63.6	

*F* forward primer, *R* reverse primer.

### Purifying and SNPs analysis

The PCR products were purified by enzyme, each containing 1.25 µL of shrimp alkaline phosphatase and 0.025 µL exonuclease I (New England Biolabs, UK) and worked at 37 °C for 30 min, 80 °C for 15 min and then subsequently storage at 4 °C. Next, the purified PCR products were sequenced using ABI PRISM 3730 DNA Analyzer (Applied Biosystems, Foster City, CA). The SNPs analysis mainly focused on the promoter region of CTLA-4 and CD28, in which we found 12 candidate SNPs in Taiwanese through searching thoroughly, including rs11571315, rs733618, rs4553808, rs11571316, rs62182595, rs16840252, rs5742909, rs1879877, rs3181096, rs3181097, rs3181098, rs56228674, and rs3116496. Additionally, the rs231775 (exon 1) and the rs3087243 (3’UTR) of CTLA-4 and the rs3116496 (intron 3) of CD28 were also selected to be candidate SNPs, because they were hot points in research about immune diseases.

### Functional analysis

The wild type reporter was constructed first. We amplified the promoter region of CTLA4 by PCR to construct the promoter-reporter that was digested with HindIII and SacI. Subsequently, the reporter was transferred to the pNL1.1 expression vector (Promega) that was with NanoLuc luciferase and transfected into DH5α competent cells. Then, the site-directed mutagenesis was used to produce the gene fragment with rs11571315 C>T by Quick-Change mutagenesis kit (Strata-gene) with the mutagenesis forward primer 5’-GCT CCT CTA CAT AAT ACT TCA ATT CCA GCA TTG-3’ and the reverse primer 5’-CAA TGC TGG AAT TGA AGT ATT ATG TAG AGG AGC-3’. And the forward primer for rs5742909 C>T was 5’-GTT ATC CAG ATC CTT AAA GTG AAC ATG AAG C-3’ and the reverse primer was 5’-GCT TCA TGT TCA CTT TAA GGA TCT GGA TAA C-3’. Next, these fragments were transferred to pNL1.1 expression vector to construct a reporter with site-directed mutation. 1 μg of the reporter and 1 μg of the pGL 4.5 [Luc2/TK] vector (Promega) were simultaneously transfected into 2.5 × 10^5^ K562 cells, where the pGL 4.5 with the Firefly luciferase was used as an internal control to offset the error of transfection efficiency. Before that, the 1 × 10^6^ K562 cells were cultured in 90% RPMI 1640 medium supplemented with 10% fetal bovine serum, penicillin (50 U/mL) and streptomycin (50 μg/mL), then they were transfected with 5 μg of allele-specific expression vector using LF2000 in the light of the manufacturer’s protocol. After culturing for 24 h, the Luciferase Assay System (Nano-Glo® Dual-Luciferase® Reporter Assay System, Promega) was used to measure the luciferase activity according to the manufacturer’s instructions.

### Statistical analysis

Statistical analysis was performed via SPSS 17.0. Allele frequency of each SNP of control was analyzed by the HWE analysis to reduce sampling bias in case–control studies. If the p-value of HWE is higher than 0.05, we can say that the control group that was enrolled in this study can represent the entire population. Genotype frequency of CTLA-4 and CD28 gene were compared between the control group and test group using chi-square test or Fisher’s exact test to appraise the association between ITP and gene polymorphism. The SNPs data were not all available, because of the insufficient DNA samples and unclear sequencing results. In the functional study, the luciferase activity was detected by the luminescence ratio of NanoLuc luciferase to Firefly luciferase (NanoLuc/ Firefly). The luminescence ratio of the wild type was corrected to 1, being a reference of RLU. And the Mann–Whitney U test was used to analyze the difference of RLU between the reporter with wild type and with site-directed mutation to evaluate the transcription level affected by SNP that was located in the promoter region of the gene.

## Supplementary Information

Supplementary Information 1.

Supplementary Information 2.

## Data Availability

The data supporting this study are included in this published article and its Supplementary Information.
