# Supplementary material for: Investigation of the correlation between immune thrombocytopenia and T cell activity-regulated gene polymorphism using functional study
Source: Sci Rep. 2022 Apr 22;12:6601. doi: 10.1038/s41598-022-10631-z (PMC9033768; doi:10.1038/s41598-022-10631-z)
Supplement: Supplementary file 2 — Supplementary Information 2. [file 41598_2022_10631_MOESM2_ESM.doc]

**Investigation of the correlation between immune thrombocytopenia and T cell activity-regulated gene polymorphism using functional study**

Ding-Ping Chen^1,2,3^, Wei-Tzu Lin^1^, Ying-Hao Wen^1,4^, Wei-Ting Wang^1^

Supplementary Figure 1. The bar chart and raw data of relative light unit of the rs11571315 C>T reporter, the rs5742909 C>T reporter, and wild type reporter.


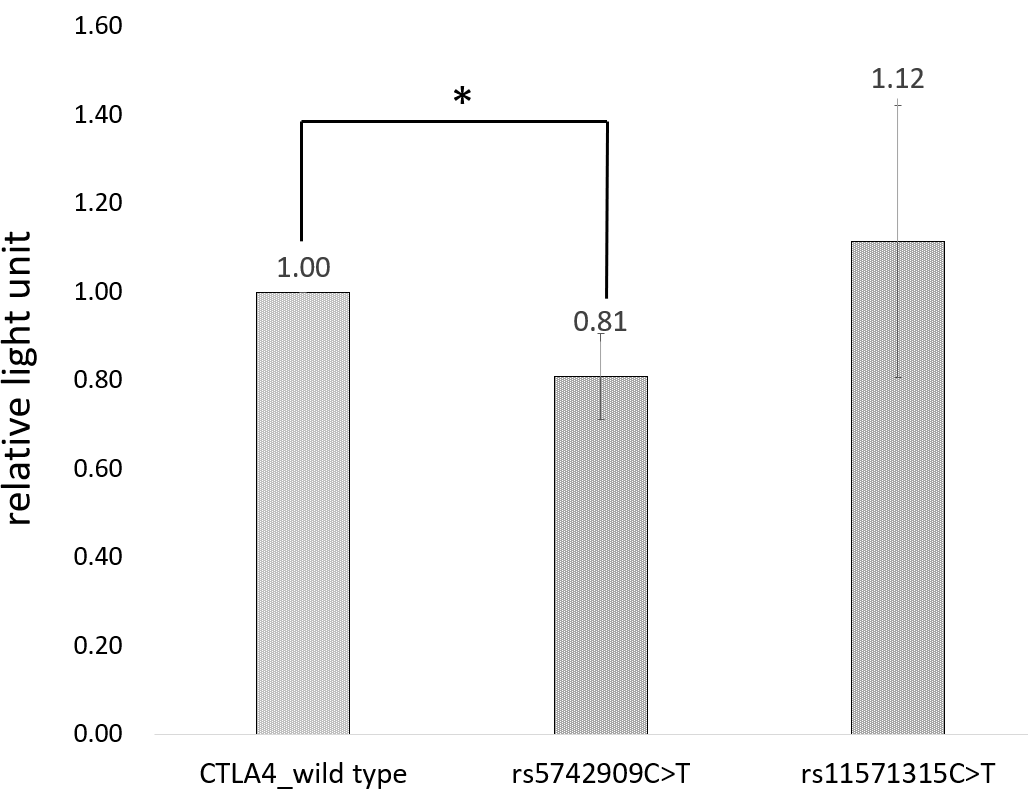

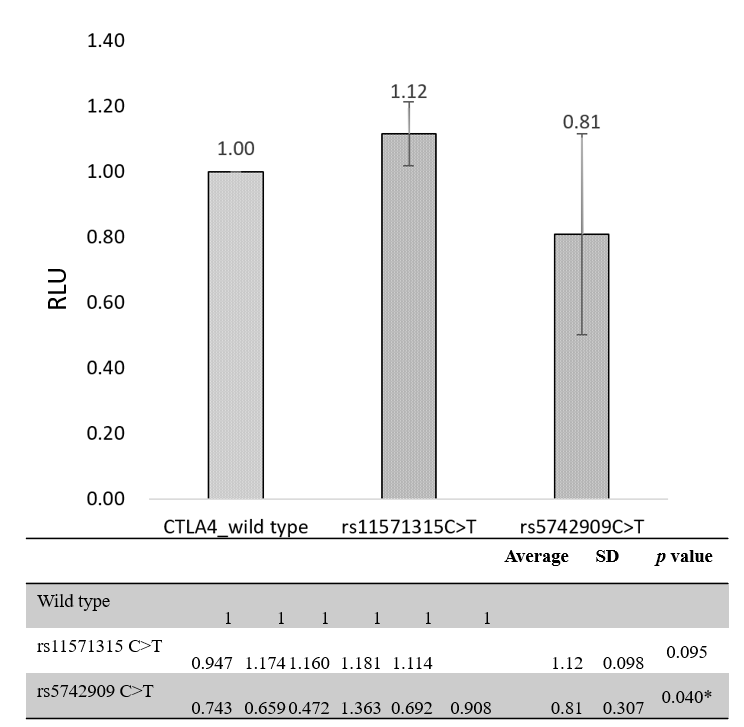


Supplementary Table 1. Statistical analysis of CD28 SNPs and ITP.

|  |  | **Genotype frequency** | | **Odds ratio** |  |
| --- | --- | --- | --- | --- | --- |
| **SNP** | **Genotype** | **Patient (n)** | **Control (n)** | **95 % CI.** | **P value** |
| **-1198_rs1879877**  T>G | GG vs. GT vs. TT |  |  |  | 0.619 |
|  | TT | 14 | 13 | Ref. | 1.000 |
|  | GT | 11 | 13 | 0.786 (0.261-2.365) | 0.668 |
|  | GG | 7 | 4 | 1.625 (0.384-6.872) | 0.721 |
|  | TT vs. GT+GG |  |  | 0.983 (0.360-2.685) | 0.974 |
|  | TT+GT vs. CC |  |  | 1.820 (0.474-6.989) | 0.379 |
| **-1066_rs3181096**  C>T | CC vs. CT vs. TT |  |  |  | 0.922 |
|  | CC | 19 | 18 | Ref. | 1.000 |
|  | CT | 10 | 10 | 0.947 (0.319-2.812) | 0.922 |
|  | TT | 3 | 2 | 1.421 (0.212-9.518) | 1.000 |
|  | CC vs. CT+TT |  |  | 1.026 (0.372-2.833) | 0.960 |
|  | CC+CT vs. TT |  |  | 1.448 (0.225-9.332) | 1.000 |
| **-1059_rs3181097**  A>G | AA vs. AG vs. GG |  |  |  | 0.122 |
|  | AA | 12 | 6 | Ref. | 1.000 |
|  | AG | 12 | 19 | 0.316 (0.093-1.067) | 0.079 |
|  | GG | 8 | 5 | 0.800 (0.181-3.536) | 1.000 |
|  | AA vs. AG+GG |  |  | 0.417 (0.133-1.310) | 0.129 |
|  | AA+AG vs. GG |  |  | 1.667 (0.478-5.817) | 0.421 |
| **-1042_rs3181098**  G>A | AA vs. AG vs. GG |  |  |  | 0.912 |
|  | GG | 18 | 18 | Ref. | 1.000 |
|  | AG | 11 | 10 | 1.100 (0.375-3.230) | 0.862 |
|  | AA | 3 | 2 | 1.500 (0.223-10.077) | 1.000 |
|  | GG vs. AG+AA |  |  | 1.167 (0.425-3.205) | 0.765 |
|  | GG+AG vs. AA |  |  | 1.448 (0.225-9.332) | 1.000 |
| **rs56228674**  C>T | CC vs. CT vs. TT |  |  |  | 1.000 |
|  | CC | 28 | 27 | Ref. | 1.000 |
|  | CT | 4 | 3 | 1.286 (0.263-6.289) | 1.000 |
|  | TT | 0 | 0 | NA | NA |
|  | CC vs. CT+TT |  |  | 1.286 (0.263-6.289) | 1.000 |
|  | CC+CT vs. TT |  |  | NA | NA |
| **17 +3_rs3116496**  T>C | CC vs. CT vs. TT |  |  |  | 0.493 |
|  | TT | 25 | 22 | Ref. | 1.000 |
|  | CT | 6 | 8 | 0.660 (0.198-2.199) | 0.497 |
|  | CC | 1 | 0 | NA | 1.000 |
|  | TT vs. CT+CC |  |  | 0.770 (0.240-2.469) | 0.660 |
|  | TT+CT vs. CC |  |  | NA | 1.000 |

95 % CI: 95% confidence interval; NA: not applicable.

p values were counted from Chi-square test or Fisher's exact test, where “*” means that it had significant difference between test group and control group.
